# Identification of Residual Blood Proteins in Ticks by Mass Spectrometry Proteomics

**DOI:** 10.3201/eid1408.080227

**Published:** 2008-08

**Authors:** Samanthi Wickramasekara, Jonas Bunikis, Vicki Wysocki, Alan G. Barbour

**Affiliations:** * University of Arizona, Tucson, Arizona, USA; † University of California Irvine, Irvine, California, USA; 1 These authors contributed equally to this article.

**Keywords:** Tick, blood, protein, mass spectrometry, proteomics, host, Ixodes, Amblyomma, reservoir, vector, dispatch

## Abstract

Mass spectrometry–based proteomics of individual ticks demonstrated persistence of mammalian host blood components, including α- and β-globin chains, histones, and mitochondrial enzymes, in *Ixodes scapularis* and *Amblyomma americanum* ticks for months after molting. Residual host proteins may identify sources of infection for ticks.

Without transovarial or venereal transmission, a vector-borne pathogen’s persistence in nature depends on successful passage between >1 species of vertebrate reservoirs. For Lyme borreliosis in eastern North America, black-legged tick (*Ixodes scapularis*) larvae acquire *Borrelia burgdorferi* from a reservoir host during their first blood meal. Infection persists through subsequent molts, and when a tick feeds for the second time as a nymph it may transmit infection to another competent reservoir or to a human. Reservoir hosts for *B*. *burgdorferi* are commonly white-footed mice but also include chipmunks, voles, shrews, and ground-foraging birds.

When a tick-borne agent has multiple reservoir hosts, assigning relative contributions of each species to maintenance of the pathogen in the environment may be difficult. One approach is to capture animals, sample blood or tissue for evidence of infection, and examine embedded ticks for the microorganism (*1*,*2*). However, this approach is labor- and resource-intensive, and sample sizes are limited. Greater statistical power could be attained with fewer resources if questing ticks were examined not only for infection but also for the source of the last blood meal because the tick would likely have acquired the infection from that vertebrate. If the tick were engorged, this would be straightforward with the PCR, as demonstrated in mosquitoes (*3*,*4*). However, host-seeking nymphal hard ticks are flat because their last blood meals were months earlier. Use of PCR to identify DNA of vertebrate mitochondria in ticks has been reported (*5*,*6*), but results lacked full sensitivity (*7*,*8*).

An alternative approach is to detect residual proteins from the blood meal. Uptake and retention of host immunoglobulin into the hemolymph of different species of ticks have been documented (*9*), and Venneström and Jensen found vertebrate actin in *I*. *ricinus* nymphs weeks after the molt (*10*). Given these observations, we hypothesized that sufficient host proteins remained in flat ticks for identification of blood meal by using proteins instead of DNA.

## The Study

We used mass spectrometry (MS)–based proteomics, as described by Breci et al. and Koller et al. (*11*,*12*). Individual ticks or pools were pulverized after freezing in liquid nitrogen. Total proteins were precipitated in 95% ethanol at –20°C and recovered by centrifugation. Proteins of individual ticks were reduced with 100 mmol/L dithiothreitol, alkylated with 50 mmol/L iodoacetamide, digested with trypsin at a final concentration of 0.01 μg/μL, and filtered through a C18 cartridge before being subjected to liquid chromatography (LC) with a 5%–50% acetonitrile gradient in 0.1% formic acid, followed by tandem MS (LC-MS/MS; LTQ ThermoElectron, San Jose, CA, USA). Proteins of pooled ticks were separated by 1-dimensional sodium dodecyl sulfate–polyacrylamide gel electrophoresis, and gel slices were digested in situ with trypsin before using LC-MS/MS. Upwards of ≈7,000 spectra were submitted to a protein identification algorithm for each pooled sample. Sizes of sequenced peptides ranged from 5 aa to 34 aa. The SEQUEST search algorithm (http://fields.scripps.edu/sequest) with data-filtering criteria was used to identify sequence matches of output against databases of rabbit, sheep, deer, goat, mouse (*Mus musculus*), and tick proteins.

We studied *I*. *scapularis* and the lone-star tick, *Amblyomma americanum*, which is a vector of human monocytic ehrlichiosis in the United States. Ticks were provided by the tick-rearing facility of the Department of Entomology and Plant Pathology of Oklahoma State University (Stillwater, OK, USA). We examined pools of 15 *A*. *americanum* nymphs that had fed on sheep or rabbits as larvae and were 3 months postmolt. Predominant vertebrate peptides in all pools were α- and β-globin chains of hemoglobin and immunoglobulins. Sequences of these proteins corresponded to the source of the blood for the ticks. Other mammalian proteins detected in pools from ticks fed on sheep or rabbits were histone H3, histone H2, mitochondrial malate dehydrogenase, glyceraldehyde-3-phosphate dehydrogenase, mitochondrial ATP synthase, interferon regulatory factor, α tubulin, β tubulin, and transferrin.

We then studied individual ticks that had fed as larvae on mice (*I*. *scapularis*), rabbits (*A*. *americanum* and *I*. *scapularis*), or sheep (*A*. *americanum*) and examined flat nymphs of *A*. *americanum* at 7 months postmolt and *I*. *scapularis* at 3–11 months postmolt. We also examined *I*. *scapularis* adults (2 males and 1 female) that had fed as larvae and nymphs on mice and were 3–5 months postmolt. Concentrations of extracted proteins from individual ticks were 50–70 μg/tick. The Figure shows representative LC-MS/MS spectra of an *A*. *americanum* nymph that had fed on a sheep as a larva. Panel A shows the tandem mass spectrum for the singly charged peptide AAVTGFWGK, corresponding to residues 8–16 of sheep hemoglobin β-subunit (P02075). Panel B shows the tandem mass spectrum for doubly charged VKVDEVGAEALGR, corresponding to residues 17–29 of the same protein. These 2 peptides cover 15.2% of the protein sequence and differ from the orthologous sequence of rabbit (P02099) at 8 of 22 positions.

**Figure:**
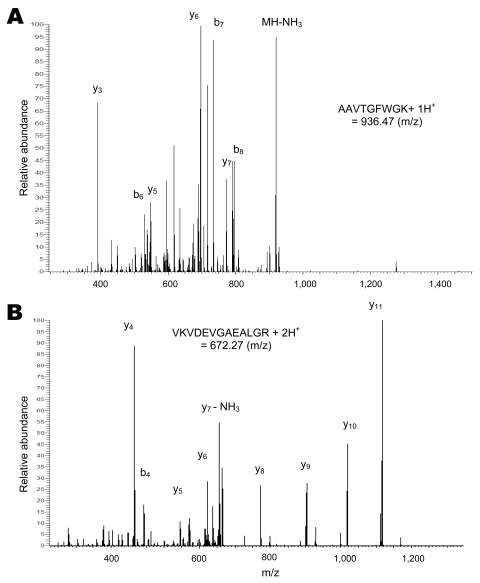
Tandem mass spectra of 2 peptides from sheep hemoglobin β-subunit identified in a nymph of an *Amblyomma americanum* tick. A) Singly protonated AAVTGFWGK. B) Doubly protonated VKVDEVGAEALGR. The peaks are labeled in the conventional manner: b ions include the N-terminus of the peptide and y ions include the C-terminus, with subscripts indicating the number of amino acid residues in the fragment.

The Table summarizes results of all individual tick analyses. There was no correlation between number of proteins detected and postmolt period. Although some proteins, such as immunoglobulin and histone H3, were detected in both species, other proteins distinguished between *A*. *americanum* and *I*. *scapularis*. Globin chains were more commonly found in *A*. *americanum* than in *I*. *scapularis* nymphs. Cytochrome c-type heme lyase, which binds heme moieties and is transported from the cytoplasm to mitochondria of eukaryotes, was present in all samples of *I*. *scapularis* but not in *A*. *americanum* (2-sided p<0.001, by likelihood ratio). Peptides detected included those specific for the host animal for the blood meal.

**Table:** Table. Vertebrate proteins detected by mass spectrometry in extracts of *Amyblomma americanum* or *Ixodes scapularis* flat ticks

Protein in tryptic digest	No. ticks in which protein was detected/no. examined						
	*A. americanum* nymphs			*I. scapularis* nymphs			*I. scapularis* adults
	Fed on rabbit	Fed on sheep		Fed on rabbit	Fed on mouse		Fed on mouse
Immunoglobulin	3/4	3/4		3/5	4/10		0/3
Globin (α or β)	1/4	3/4		0/5	0/10		3/3
Histone H3	1/4	3/4		3/5	5/10		3/3
Histone H2	3/4	3/4		4/5	6/10		3/3
Tubulin	1/4	2/4		5/5	9/10		3/3
Keratin	0/4	3/4		5/5	7/10		1/3
Actin	1/4	3/4		5/5	10/10		0/3
Cytochrome c-type heme lyase	0/4	0/4		5/5	10/10		3/3
>1 of above	4/4	4/4		5/5	10/10		3/3

## Conclusions

Digestion of a blood meal in ticks differs from what generally occurs in hematophagous insects. In ticks, digestion takes place gradually within cells of the intestinal tract after endocytosis, rather than by intraluminal enzymatic breakdown of blood cells and plasma components, as in insects (*13*). The combination of slow assimilation and uptake of some host proteins into hemolymph may explain persistence of host blood proteins for months after feeding and molting. Immunoglobulins in hemolymph have been demonstrated, but demonstration of several other proteins, including globin chains, histones, and mitochondrial enzymes, indicates that host protein persistence is not limited to 1 type of molecule.

The success of our study depended on access to a large database of protein sequences for sheep, rabbits, and laboratory mice. For most host species for these ticks in nature, such as the white-footed mouse (*Peromyscus leucopus*), protein databases currently are much less extensive. Thus, a priority before application of this approach is MS-based sequencing of highly prevalent proteins from the blood of *P*. *leucopus* and other host species. Although LC-MS/MS of individual ticks is feasible and highly sensitive, its cost confines it to exploratory studies. High-throughput analysis of hundreds or thousands of specimens will likely require species-specific assays that use antibodies or aptamers for detection and identification of selected proteins.

Understanding contributions of different vertebrate hosts to pathogen maintenance is a prerequisite for effective monitoring, modeling, and disease prevention efforts that focus on natural reservoirs. Unfortunately, this level of understanding has not been broadly achieved. A major impediment to success in this area for most tick-borne zoonoses has been the absence of reliable and reproducible methods for identification of the vertebrate source of the infection for the tick vector by characterizing residual blood components. Our study shows a way to achieve this goal. Similar data on uninfected ticks would establish the denominator for prevalence studies and indicate the relative competence of different host species.
